# Clinical and multimodal imaging features of adult-onset neuronal intranuclear inclusion disease

**DOI:** 10.1007/s10072-024-07699-y

**Published:** 2024-07-18

**Authors:** Rui Zhu, Junyu Qu, Guihua Xu, Yongsheng Wu, Jiaxiang Xin, Dawei Wang

**Affiliations:** 1 Department of Radiology, Qilu Hospital of Shandong University, Jinan, 250012 China; 2 MR Research Collaboration, Siemens Healthineers Ltd, Shanghai, China; 3 Qilu Medical Imaging Institute of Shandong University, Jinan, 250012 China; 4 Shandong Key Laboratory: Magnetic Field-free Medicine & Functional Imaging (MF), Jinan, 250012 China

**Keywords:** Neuronal intranuclear inclusion disease, Clinical manifestations, Multimodal imaging, Neuroimaging biomarkers

## Abstract

**Objectives:**

This study aimed to analyze the clinical and multimodal imaging manifestations of adult-onset neuronal intranuclear inclusion disease (NIID) patients and to investigate NIID-specific neuroimaging biomarkers.

**Methods:**

Forty patients were retrospectively enrolled from the Qilu Hospital of Shandong University. We analyzed the clinical and imaging characteristics of 40 adult-onset NIID patients and investigated the correlation between these characteristics and genetic markers and neuropsychological scores. We further explored NIID-specific alterations using multimodal imaging indices, including diffusion tensor imaging (DTI), magnetic resonance spectroscopy (MRS), and brain age estimation. In addition, we summarized the dynamic evolution pattern of NIID by examining the changes in diffusion weighted imaging (DWI) signals over time.

**Results:**

The NIID patients’ ages ranged from 31 to 77 years. Cognitive impairment was the most common symptom (30/40, 75.0%), while some patients (18/40, 45.0%) initially presented with episodic symptoms such as headache (10/40, 25.0%). Patients with cognitive impairment symptoms had more cerebral white matter damage (χ2 = 11.475, *P* = 0.009). The most prevalent imaging manifestation was a high signal on DWI in the corticomedullary junction area, which was observed in 80.0% (32/40) of patients. In addition, the DWI dynamic evolution patterns could be classified into four main patterns. Diffusion tensor imaging (DTI) revealed extensive thinning of cerebral white matter fibers. The estimated brain age surpassed the patient’s chronological age, signifying advanced brain aging in NIID patients.

**Conclusions:**

The clinical manifestations of NIID exhibit significant variability, usually leading to misdiagnosis. Our results provided new imaging perspectives for accurately diagnosing and exploring this disease’s neuropathological mechanisms.

**Supplementary Information:**

The online version contains supplementary material available at 10.1007/s10072-024-07699-y.

## Introduction

Neuronal intranuclear inclusion disease (NIID) is a rare progressive neurodegenerative condition characterized by widespread eosinophilic intranuclear inclusions in central, peripheral, and autonomic nervous system cells and visceral organs [1–3]. The clinical manifestations are significantly heterogeneous and can be classified into three types based on the main symptoms: central, peripheral, and autonomic nervous system symptoms [4]. NIID is categorized into infantile, adolescent, and adult types, with the adult NIID type categorized into familial and disseminated types based on genetic characteristics [1].

However, clear diagnostic criteria for NIID have yet to be established. There are three main bases for clinical diagnosis, including the characteristic high diffusion-weighted imaging (DWI) signal in the corticomedullary junction area of cranial MRI [4], eosinophilic intranuclear inclusion bodies found in skin biopsy [2], and abnormal amplification of the GGC sequence in the 5’ UTR of the NOTCH2NLC gene [5]. In the diagnostic process proposed by Sone [4], the high signal at the corticomedullary junction on DWI imaging is used as the strongest and most readily available evidence guiding further skin biopsy and diagnosis. Therefore, early recognition of NIID imaging features is significant.

Currently, comprehensive research on NIID is still lacking. Since the first report in 1968 [3], there have been less than 700 reported cases in the literature [6], of which fewer than 100 have complete imaging manifestations [7]. In particular, few studies have addressed multimodal imaging and dynamic changes in adult-type NIID patients.

This study aimed (1) to analyze the clinical and imaging manifestations of 40 adult-onset NIID patients to provide vital information for early and accurate diagnosis of the disease; (2) to investigate NIID-specific neuroimaging biomarkers by analyzing multimodal imaging findings and chronologically varying dynamic MRI evolution patterns, and (3) to investigate the factors influencing white matter hyper-signalization in NIID patients and to produce new imaging perspectives on the neuropathological mechanisms of this disease.

## Methods

### Subjects

Forty patients (15 males and 25 females, aged between 31 and 77 years) were retrospectively enrolled from the Qilu Hospital of Shandong University between January 2019 and January 2024. The patients were consisted of 3 patients from one pedigree and 37 individuals affected sporadically. Detailed information was obtained from each patient, the patient’s family, or clinical records. Information regarding disease progression, family history, pathological findings from skin biopsy, genetic data, and other clinical manifestations was collected. The patients were diagnosed with NIID by skin pathology and/or analysis of the NOTCH2NLC gene (*n* = 30) or clinical diagnosis without pathological and genetic testing (*n* = 10). All individuals were evaluated by two or more neurologists. The summary of sample sizes and main findings for each examination with NIID patients was shown in Supplemental Table 1.

### Imaging protocols

Magnetic resonance imaging (MRI) was performed on a Siemens Magnetom Verio 3.0 Tesla scanner (Siemens Erlangen, Germany) with a standard 20 Ch-receive head/neck coil. During the scanning process, participants were instructed to keep their eyes closed and remain still. MRI scanning, including conventional T1-weighted Fluid-attenuated Inversion Recovery (T1-FLAIR), fast spin‒echo T2-weighted imaging (T2WI), fluid-attenuated inversion recovery (FLAIR), and diffusion-weighted imaging (DWI), was performed for all patients. Then, each patient’s Fazekas grade was categorized into three severity groups [8] based on FLAIR imaging.

Overall, 21 patients underwent 3D-T1WI sagittal high-resolution and diffusion tensor imaging (DTI) sequences, and twenty healthy individuals matched for age, and gender were also collected as the control group. The demographic information of both 21 patients with NIID and 20 healthy controls was shown in Supplemental Table 2. Anatomical T1-weighted reference images were collected with an MPRAGE sequence and the following parameters: TE = 2.3 ms, TR = 2000 ms, resolution = 1 × 1 × 1 mm^3^, matrix size = 192 × 256 × 256, Inversion Time (TI) = 900 ms, iPAT = 2, bandwidth = 190 Hz, and total acquisition time = 4 min 40 s. DTI images were acquired in the axial plane using the single shot-echo planar imaging technique with the following parameters: b values = 0, 1000 s/mm2; diffusion direction = 64; TR/TE = 6400/98 ms; FOV = 256 × 256 mm^2^; resolution = 2 × 2 mm^2^; slices = 45, slice thickness = 3 mm; bandwidth = 1502 Hz, and total acquisition time = 7 min 17 s. An analytical approach to DTI using tract-based spatial statistical analysis (TBSS) to assess changes in cerebral white matter fiber bundles in patients with NIID (more details in Supplemental Material).

Five patients underwent proton magnetic resonance spectroscopy (^1^H MRS) scans. Single-voxel ^1^H magnetic resonance spectra were acquired using the point-resolved spectroscopy (PRESS) sequence. These spectra were obtained from a volume of interest (VOI) measuring 12 × 16 × 10 mm^3^, precisely positioned in the corticomedullary junction area corresponding to the site of the high-intensity DWI signal. The parameters for the PRESS sequence were as follows: TR/TE = 1500/136 ms, averages = 300, vector size 1024 points, flip angle = 90°, acquisition duration = 853 ms, acquisition bandwidth = 1200 Hz, and water suppression bandwidth = 50 Hz. We also collected spectral data from the same voxel locations in five healthy individuals matched for age and gender as the control group. The learning-compression model (LC-Model) algorithm was used to fit the experimental data in the frequency domain and to quantify the ^1^H MRS metabolites. Furthermore, nineteen patients underwent two or more MRI scans, with follow-up ranging from two to five years.

### Brain age estimation

Brain age was estimated using the BrainAgeR analysis pipeline (v2.1) developed by Cole and colleagues [9–11] and based on structural three-dimensional T1-weighted images. Brain-predicted age, henceforth referred to as brain age, is determined through a voxel-based analysis of regional volume differences, a method fine-tuned for accuracy in training and future application with extensive cohort studies [12]. A pre-trained Gaussian regression model, part of the Kernlab package in R, facilitated brain age estimation through machine learning. The process begins with the segmentation of T1-weighted scans into gray and white matter components, then advances to normalization via nonlinear spatial alignment, employing the DARTEL toolbox of SPM12 [13]. Subsequent quality control checks were conducted on images processed with a modified FSL slicesdir tool at every stage. Cerebrospinal fluid (CSF) was removed, and the probabilistic tissues in the gray and white matter were vectorized, combined, and subjected to principal component analysis (PCA) to lower data complexity. Only components accounting for the top 80% variance were selected for brain age prediction. The concept of the brain age gap highlights the discrepancy between an individual’s chronological age and their estimated brain age. To assess this feature, we determined the relative difference [14] for each study participant by using the formula: *(brain age estimation - chronological age) / chronological age*. This calculation indicates whether an individual’s brain aging is progressing faster or slower than expected based on their chronological age, offering a proportionally adjusted perspective. A positive outcome suggests that the estimated brain age surpasses the participant’s actual age, signifying advanced brain aging. Conversely, a negative result implies that the participant’s actual age exceeds their estimated brain age, indicating delayed brain aging.

### Clinical assessment

Neurologists performed detailed neurological examinations. Cognitive function was evaluated using the Mini-Mental State Examination (MMSE) and the Montreal Cognitive Assessment (MoCA).

### Statistical analyses

The descriptive statistical analysis used frequencies and percentages (n (%)) for the categorical variables and medians (ranges) for numerical variables. All quantitative data were tested for normality using the Shapiro-Wilk test. T-tests were used for normally distributed data. For non-normally distributed data, we performed Mann‒Whitney U or Kruskal‒Wallis tests. For categorical variables, we employed the χ^2^ test.

We investigated the potential association between the size of the NOTCH2NLC GGC repeat expansions and various factors including age, neuropsychological assessment scores, and the presence/absence of certain clinical manifestations. Furthermore, we examined the effects of the number of gene repeats, MMSE score, MoCA score and various clinical manifestations on cerebral white matter hyperintensity in NIID patients. All correlations were analyzed using Spearman’s rank. Statistical significance was defined as *P* < 0.05. Last, data analysis was performed using SPSS 26.0 statistical software.

## Results

### Clinical features of the NIID patients

In this study, we classified the clinical symptoms of NIID patients into distinct categories based on their primary manifestations, as outlined in Table 1. Symptoms varied from single to multiple presentations across individuals. Specifically, cognitive impairment was observed in 30 patients, with 10 reporting it as their initial symptom. Idiopathic tremor was the initial diagnosis in three cases. Notably, nearly half of the study participants experienced paroxysmal symptoms, with episodic headaches accompanied by nausea and vomiting being the most prevalent. Furthermore, autonomic dysfunction was significant, with up to 20% of patients experiencing dysuria or urinary incontinence. In some cases, these autonomic symptoms preceded central nervous system manifestations by several years.

**Table 1:** Demographic and clinical manifestation of patients with NIID (*n* = 40)

Variables	*n* (%) or median (range)
Gender ratio (male/female)	15/25
Age at diagnosis (years)	63 (31–77)
< 60	15 (37.5%)
≥60	25 (62.5%)
MMSE (scores) ^a^	21 (6–29)
MoCA (scores) ^b^	14 (4–28)
Expanded NOTCH2NLC GGC repeat size ^b^	110.5 (91–191)
Cognitive impairment	30 (75.0%)
Movement disorders	21 (52.5%)
Tremor	12 (30.0%)
Ataxia	10 (25.0%)
Bradykinesia	6 (15.0%)
Dysphagia	3 (7.5%)
Limb weakness	9 (22.5%)
Paroxysmal symptoms	18 (45.0%)
Headache	10 (25.0%)
Disturbance of consciousness	8 (20.0%)
Vomiting	8 (20.0%)
Abnormal mental behaviors	4 (10.0%)
Pyrexia	2 (5.0%)
Abdominal pain	1 (2.5%)
Encephalitic episodes	1 (2.5%)
Stroke-like episodes	1 (2.5%)
Epilepsy	1 (2.5%)
Autonomic dysfunction	8 (20.0%)
Bladder dysfunction	7 (17.5%)
Miosis	1 (2.5%)
Other symptoms	12 (30.0%)
Dizzy	7 (17.5%)
Visual disturbance	3 (7.5%)
Hearing loss	2 (5.0%)
Sleep disorder	2 (5.0%)

^a^12 patients had no data on MMSE; ^b^12 patients had no data on MoCA; ^c^16 patients had no data on NOTCH2NLC GGC repeat size

MMSE: Mini-Mental State Examination; MoCA: Montreal Cognitive Assessment

### Neuropsychological assessment of the NIID patients

Cognitive dysfunction was commonly the initial NIID patient symptom. In our study, a total of 28 individuals underwent MMSE and MoCA examinations (Table 1). The MMSE and MoCA scores were 21 (range 6–29) and 14 (range 4–28), respectively. Most (27/28, 96.4%) NIID patients exhibited varying degrees of cognitive decline (MMSE < 27 and/or MoCA < 26), and as many as two-thirds (19/28, 67.8%) fulfilled the diagnostic criteria for dementia (MMSE ≤ 24).

### Genetic and skin biopsy analyses of the NIID patients

The median size of the expanded NOTCH2NLC GGC repeats in the 24 NIID patients was 110.5 (range 91–191). Skin biopsies obtained from 19 of these patients revealed eosinophilic intranuclear inclusions in the fibroblasts and sweat gland cells of 17 patients. Remarkably, one patient displayed visible eosinophilic intranuclear inclusion bodies in the skin biopsy despite having a normal GGC repeat count of 19. Furthermore, in a familial cluster involving three patients, the mother exhibited both abnormal GGC repeat expansions (131 repeats) and eosinophilic intranuclear inclusions in sweat gland cells. In contrast, her two sons had significantly larger expansions (> 158 and > 161 repeats, respectively) but showed neither clinical nor pathological signs of NIID.

We found that patients who were younger at diagnosis had longer GGC repeat expansions (*r* = -0.422, *P* = 0.043). Moreover, a negative correlation was discovered between the NOTCH2NLC GGC repeat expansions sizes and MoCA scores (*r* = -0.521, *P* = 0.032). Furthermore, the correlation analysis confirmed that GGC repeat sizes were negatively correlated with autonomic dysfunction (*r* = -0.527, *P* = 0.008), but there were no statistically significant differences in other clinical symptoms (Table 2).

**Table 2:** Relationship between the expanded NOTCH2NLC GGC repeats sizes and clinical features (*n* = 24)

Characteristic	Parameter	Size of the expanded NOTCH2NLC GGC repeats	
		Median (range)	*P* value*
Age(years)	< 60	118 (100–191)	**0.040**
	≥ 60	101.5 (91–159)	
MMSE ^a^		23 (11–29)	0.148
MoCA ^b^		14 (7–28)	**0.032**
Cognitive impairment	With this feature	115 (91–191)	0.735
	Without this feature	101 (91–161)	
Movement disorder	With this feature	110.5 (91–191)	0.798
	Without this feature	110 (91–161)	
Muscle weakness	With this feature	100.5 (91–191)	0.559
	Without this feature	113.5 (91–161)	
Paroxysmal symptom	With this feature	107.5 (91–138)	0.315
	Without this feature	118 (91–191)	
Autonomic dysfunction	With this feature	96 (91–105)	**0.008**
	Without this feature	121 (91–191)	

* Statistical significance at *P* < 0.05

^a^7 patients had no data on MMSE; ^b^7 patients had no data on MoCA

MMSE: Mini-Mental State Examination; MoCA: Montreal Cognitive Assessment

### Brain MRI features of the NIID patients

**Fig. 1:**
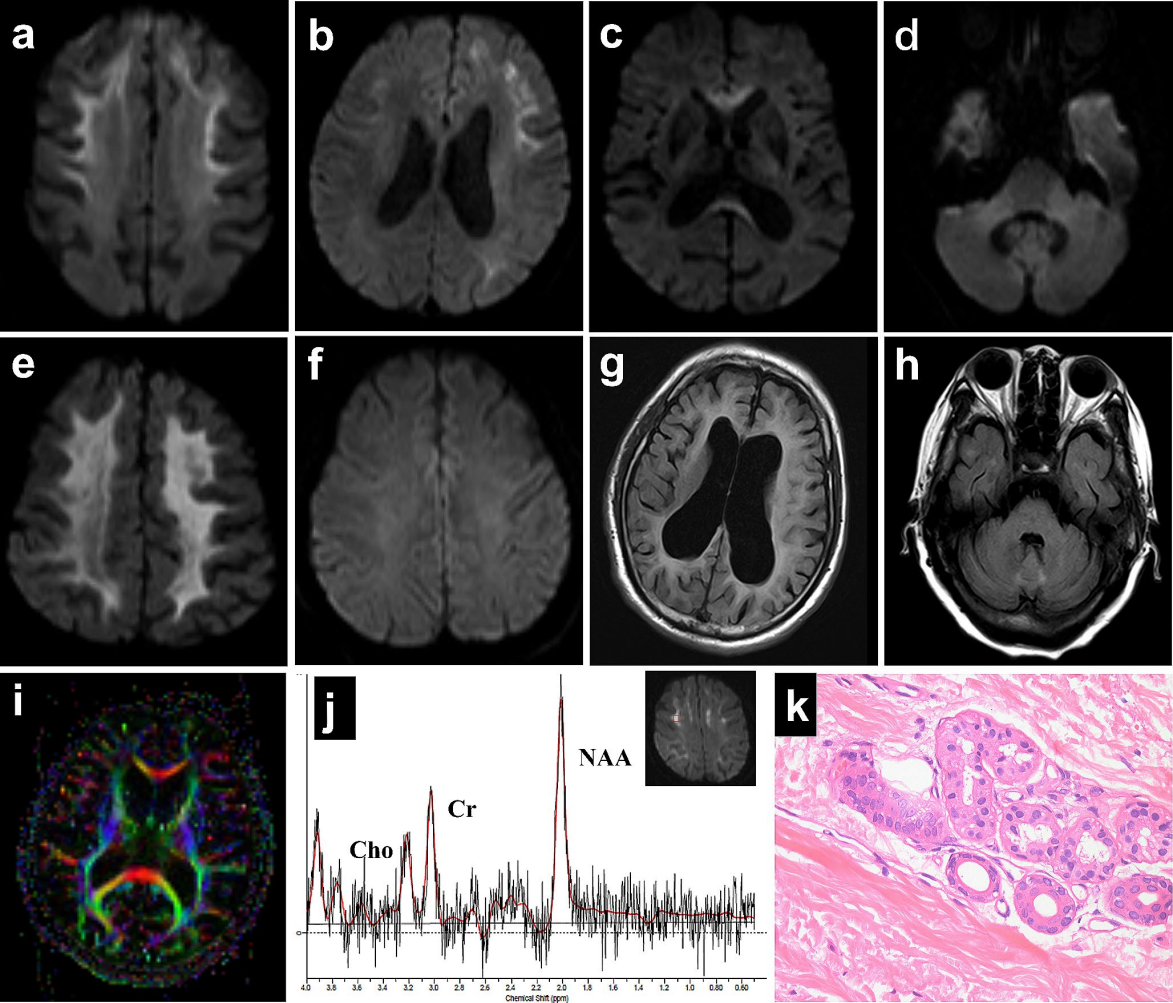
Brain MRI findings and pathological features of neuronal intranuclear inclusion disease (NIID) patients. Diffusion-weighted imaging (DWI) showed symmetric (**a**) or asymmetric (**b**) subcortical lesions at the corticomedullary junction and high intensity in the corpus callosum (**c**) and middle cerebellar peduncle (**d**). (**e**) Diffuse high-intensity DWI signal in the white matter was observed in one NIID patient. (**f**) One NIID patient did not show the typical high signals on DWI. Severe leukoencephalopathy and cerebral atrophy were detected using FLAIR (**g**) imaging. (**h**) High signal in the cerebellar earthworm on FLAIR images. (**i**) Diffusion tensor imaging (DTI) revealed extensive thinning of the cerebral white matter fibers. (**j**) Magnetic resonance spectroscopy (MRS) revealed no significant abnormalities. (**k**) Skin biopsy with HE staining (×400) showing scattered eosinophilic inclusion bodies in the nuclei of alveolar cells of small sweat glands

The result of first routine brain MRI of 40 NIID patients was shown in Table 3. The most characteristic imaging manifestation of NIID was the presence of a curvilinear high signal in the corticomedullary junction area on DWI (Fig. 1a-b). A total of 80.0% (32/40) of the patients presented with a typical subcortical lace sign, which was most commonly observed in the frontal lobes. Most cases (29/32, 90.6%) presented with a symmetrical distribution, extending along the subcortex without deep white matter involvement. In three NIID patients, an asymmetric high signal intensity was observed in one frontal lobe. In addition, two patients showed high DWI signals in extensive white matter areas (Fig. 1e). DWI hyperintensity was also observed in the genu or splenium of the corpus callosum (Fig. 1c) in half of the patients (22/40, 55.0%). Abnormally high signal intensities were also detected in the cerebellum, including the parieto-cerebellar and mid-cerebellar hemispheres, in 37.5% (15/40) of patients (Fig. 1d). Notably, two patients exhibited high signals only in the cerebellum on DWI scans without any other abnormal imaging findings. On T2WI and/or FLAIR, 50.0% of patients demonstrated symmetrical diffuse white matter hyperintensity, particularly involving the radial crown and centrum semiovale, with a Fazekas score of 3. Some patients showed high signal intensity on FLAIR images specific to the corpus callosum pressure (24/40, 60.0%), corpus callosum knee (20/40, 50.0%), and cerebellum (7/40, 17.5%) (Fig. 1h). Last, cerebral atrophy and enlargement of the supratentorial ventricles were observed (Fig. 1g).

**Table 3:** Brain MRI characteristics and dynamic changes of patients with NIID

Characteristic	All patients (*n* = 40)	Serial follow-up MRI (*n* = 19)	
		First MRI	Last MRI
**WHM in FLAIR images**			
Frontal subcortical region	35 (87.5%)	19 (100%)	19 (100%)
Parietal subcortical region	31 (77.5%)	15 (78.9%)	15 (78.9%)
Occipital subcortical region	31 (77.5%)	15 (78.9%)	16 (84.2%)
Temporal subcortical region	25 (62.5%)	11 (57.9%)	13 (68.4%)
Genu of corpus callosum	20 (50.0%)	10 (52.6%)	11 (57.9%)
Splenium of corpus callosum	24 (60.0%)	12 (63.2%)	13 (68.4%)
Cerebellum	7 (17.5%)	2 (10.5%)	2 (10.5%)
**Hyperintense lesions in DWI**			
Frontal lobe CMJ	33 (82.5%)	17 (89.5%)	17 (89.5%) 11 (57.9%)
Parietal lobe CMJ	25 (62.5%)	10 (52.6%)	
Occipital lobe CMJ	28 (70.0%)	13 (68.4%)	15 (37.5%)
Temporal lobe CMJ	26 (65.0%)	12 (63.2%)	14 (73.7%)
Genu of corpus callosum	15 (37.5%)	6 (31.6%)	6 (31.6%)
Splenium of corpus callosum	19 (47.5%)	11 (57.9%)	12 (63.2%)
Cerebellum	15 (37.5%)	5 (10.5%)	5 (10.5%)

CMJ: Corticomedullary Junction; WMH: White Matter Hyperintensity

### Altered functional MRI in patients with NIID

The DTI analysis revealed that patients with NIID exhibit extensive damage to white matter fiber bundles, including U-shaped fibers, the corpus callosum, and pyramidal tracts (Fig. 1i, Supplemental Fig. 1). Five patients’ ^1^H MR spectra showed no significant abnormal metabolism compared to healthy controls (Fig. 1j), appearing as high signal intensity on DWI in the corticomedullary junction area.

### Dynamic MRI changes in the NIID patients

**Fig. 2:**
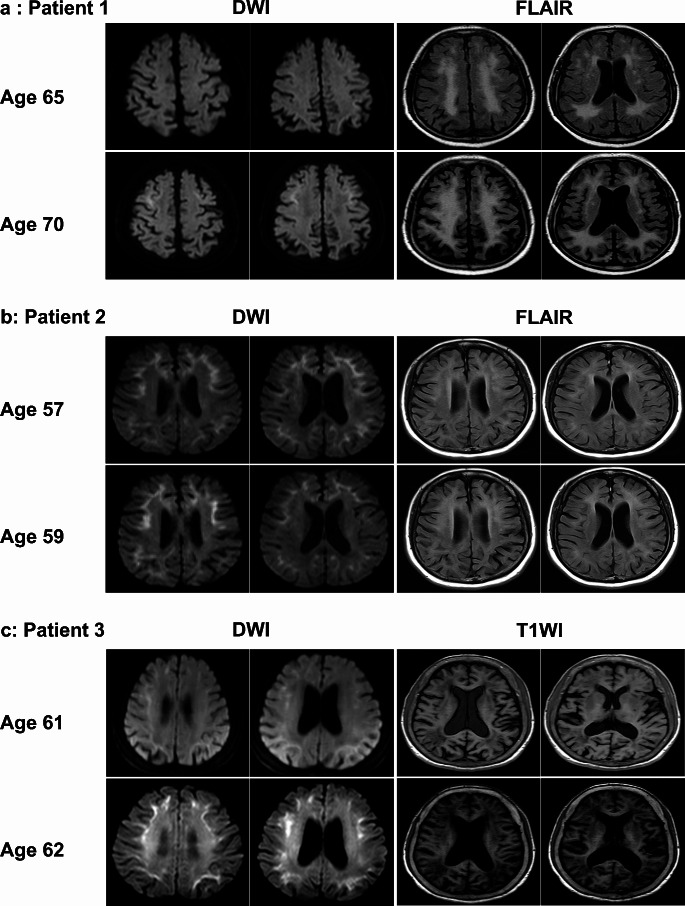
Dynamic brain imaging changes in three patients with neuronal intranuclear inclusion disease (NIID). Patient 1 (**a**) with NIID showed typical DWI hyperintensity in the bilateral frontal corticomedullary junction area at five-year follow-up, with further extension of white matter hyperintensity on FLAIR. On DWI, the high signal in the left temporal lobe disappeared after two years in patient 2 (**b**). Serial MR images of patient 3 (**c**) show an anterior to posterior propagation pattern of curvilinear hyperintense signals on DWI and apparent cerebral atrophy on T1WI

Dynamic MRI changes were evaluated in nineteen NIID patients (Table 3). Eight patients with NIID exhibited varying degrees of alterations in the abnormally high signal intensities on DWI, in which the signal mainly showed persistence and gradual progression with disease progression (Fig. 2a and c). Interestingly, signals disappeared in several cases during follow-up (Fig. 2b). The remaining eleven patients showed no significant MRI changes during follow-up.

### Advanced brain aging observed in NIID patients

**Fig. 3:**
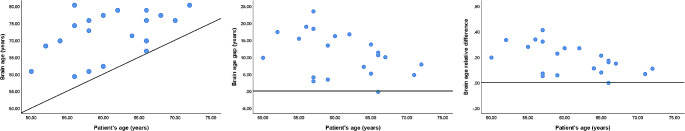
Relationships between brain age and chronologic age in patients with neuronal intranuclear inclusion disease (NIID)

The estimated brain age difference ranged from 0.26 years younger to 23.41 years older than the patient’s chronologic age. The mean brain age gap of the NIID patients was 10.92 ± 6.33 years, while the relative difference was 0.18 ± 0.11. There was a significant relationship between patient chronologic age and estimated brain age (*r* = 0.517, *P* = 0.016). Both the brain age gap and the relative difference indicated that the predicted brain age was older than the actual age of the NIID patients (both *P* < 0.001) (Fig. 3). Moreover, the disparities in both the brain age gap and the relative difference were significantly greater in patients with NIID compared to healthy controls (both *P* < 0.001) (Supplemental Fig. 2). There were no statistically significant differences between the brain age gap and the size of the abnormal gene repeat amplifications, neuropsychological scores, or clinical symptoms in NIID patients.

### Factors influencing white matter hyperintensity in NIID patients

In our study, only the presence of cognitive impairment had a significant effect on the Fazekas grade of the NIID patients (χ^2^ = 11.475, *P* = 0.009), demonstrating that patients with cognitive impairment symptoms had a higher degree of cerebral white matter damage. Other factors were not significantly associated with the Fazekas grade.

## Discussion

In this study, we comprehensively described the clinical and imaging characteristics of adult NIID patients, focusing on multimodal imaging features, including brain age, functional magnetic resonance and dynamic brain imaging changes over time, as well as factors that influence cerebral white matter hyperintensity in NIID patients.

NIID is a highly heterogeneous disease with variable, nonspecific clinical manifestations. The disease can be classified into exacerbation and chronic progression based on the onset of symptoms. Our study revealed that cognitive impairment was the most common manifestation, consistent with previous research [4], and two-thirds of people with cognitive impairment could be diagnosed with dementia. The MoCA was more sensitive in identifying cognitive impairment in NIID patients. Cognitive impairment tends to be chronic [15], but rapid cognitive decline or significant cognitive function deterioration after episodic encephalitis has also been reported [16]. Patients with Parkinson’s-like symptoms are more likely to exhibit a combination of tremor, ataxia, and slowness of movement. Chen [17] conducted genetic testing on fifteen idiopathic tremor families and discovered that sixteen patients from one family had a GGC repeat amplification mutation in the NOTCHH2NLC gene, which led to NIID diagnosis as a family line, with the suggestion that tremor might be an early differential symptom of NIID. In our study, half of the patients presented with paroxysmal symptoms. Headache accompanied by nausea and vomiting, which can be an initial symptom of NIID [18], is frequently misdiagnosed due to its lack of specificity. Additionally, bladder dysfunction is the most common autonomic symptom of NIID. Urinary incontinence may develop 6–8 years before the onset of cognitive symptoms. Most patients permitted indwelling catheters at a time when cognitive impairment was not apparent [4, 19, 20], and it is hypothesized that urinary incontinence may be due to the presence of extensive autonomic ganglia of the peripheral nervous system and intranuclear inclusions deposited in the smooth muscle cells of visceral organs [21]. Furthermore, we documented cases of visual impairment and hearing loss, which have been infrequently mentioned in previous NIID studies. Patients may present with a variety of clinical manifestations simultaneously. Some patients with a paroxysmal symptom dominant phenotype may later develop new symptoms. Therefore, a thorough and careful history is essential in the clinical management of NIID.

The amplification of the GGC sequence in the 5’ UTR of the NOTCH2NLC gene is associated with NIID pathogenesis [5, 19, 20]. Unaffected adults have no more than 40 repeat amplifications of the GGC sequence, and pathogenicity occurs when the number of repeats exceeds 60 [22]. However, some NIID patients do not exhibit GGC repeat amplification. In our study, eosinophilic intranuclear inclusion bodies were found in the skin biopsy of a patient with NIID, but no abnormal GGC repeat sequences were detected. In 2020, Jedlickova [23] diagnosed a male child with NIID at autopsy whose NOTCH2NLC sequencing did not reveal a GGC repeat amplification in the gene. This finding suggests that GGC repeat amplification mutations in the NOTCH2NLC gene may not be the only genetic cause of NIID. In addition, asymptomatic carriers of GGC with repeat amplification of the NOTCH2NLC gene exist. Deng [24] performed whole-genome sequencing on two NIID patients and their immediate relatives from different families and found that the fathers of the two patients carried a sizable number of GGC repeat amplifications of the NOTCH2NLC gene without any clinical or pathological manifestations, which is similar to the family cases in this study. It is suggested that there may be asymptomatic carriers of GGC with repeated amplification of the NOTCH2NLC gene.

The most typical imaging feature in NIID patients is a high signal intensity confined to subcortical U-fibers on DWI [4], known as the subcortical “ribbon sign”. However, in patients with disseminated NIID, isolated cases without this abnormally high signal exist [5, 19, 20]. In addition, there are rare cases in which the high-intensity DWI signal extends over a wide range of subcortical areas [6]. Yokoi [25] observed pathological changes in a patient with NIID at autopsy, finding multiple focal spongiform changes in the DWI hyperintense area. Many intranuclear inclusions were present in the cerebral white matter and cortex but were rare in the spongiform tissue. Cerebral white matter lesions are another major imaging manifestation of NIID [26]. They are mostly bilateral and have a diffuse, symmetrical distribution throughout the white matter, particularly in the frontal lobes [27]. These lesions are associated with white matter dementia in NIID patients [4]. The pathological changes seen include diffuse myelin and axonal deficits in neurons with extensive intranuclear inclusion body deposition. The corpus callosum lesions typically exhibit abnormally high signals on DWI in the knee or compression part of the corpus callosum. In some cases, foci involving only the corpus callosum may appear earlier than those involving the corticomedullary junction area. Both callosal contact fibers and subcortical arcuate fibers may be projection fibers with similar susceptibility [28]. In addition, high signal intensity in FLAIR images is visible in the middle cerebellar peduncle and cerebellar vermis, indicating that characteristic lesions in the cerebellum could serve as early diagnostic indicators for NIID [29]. The DTI examination conducted in this study revealed extensive white matter fiber disorders throughout the brain, which may be associated with the loss of myelinated nerve fibers in the brain’s white matter in NIID [25]. As reported in the literature, DTI may be more sensitive than DWI for detecting this type of white matter involvement [28]. Five patients’ ^1^H MRS examination in this study revealed no abnormalities, which is consistent with previous reports [30]. It is hypothesized that the corticomedullary junction brain region, which exhibits high signal intensity on DWI, may only show spongy degeneration in the early stages of the disease without any obvious functional neuronal impairment.

Previously, it was believed that the DWI subcortical high signal in NIID patients was constant and would not disappear [4]. However, five years of follow-up by Kawarabayashi [31] revealed that the high DWI signal in NIID patients had diminished, suggesting that this may be related to subsequent neuronal loss and glial cell proliferation. Based on our longitudinal observations, we found that there may be four main dynamic patterns of change on DWI. First, high signal intensity in the corticomedullary junction remained negative on DWI over years of follow-up. Second, DWI was initially negative but subsequently showed a typically high signal in the corticomedullary junction area. Third, the high signal intensity disappeared during follow-up. Finally, the high signal on DWI was initially located primarily in the corticomedullary junction area of the frontoparietal temporal lobe and extended to the posterior part of the brain as the disease progressed. What’s more, our findings indicate the diagnosis of NIID should be considered in patients who experience recurrent encephalitis-like episodes without apparent abnormalities on MRI in the actual phase. Therefore, it is not accurate to rely on the “ribbon sign” alone for diagnosis; rather, it should be combined with other imaging features and examinations to make a comprehensive diagnosis.

The concept of brain age is a modern approach to objectively assess changes in the brain, allowing the estimation of age-related changes in brain tissue volume independent of chronological age [32]. Predictions of brain age have been applied to several neurological disorders [33–36], and previous studies [34] have shown that brain aging is an essential factor associated with cognitive decline in adults. However, no studies have reported on brain age in patients with NIID. In our study, by calculating the difference between predicted biological and actual age, and the relative difference, we found that NIID patients showed significant premature brain aging. We hypothesized that there is an accumulation of deleterious changes in the brains of NIID patients, which may lead to changes in brain function, increasing the risk of the disease and possibly affecting prognostic recovery. The relationship with neuropsychological scores was not found in this study, possibly due to the small sample size of this study. In the future, more cases and longitudinal data are needed to investigate whether brain age is associated with clinical manifestations and prognostic recovery in NIID patients.

This study had several limitations. First, the cohort of NIID patients was quite limited in size, and neuropsychological assessment data were incomplete for some patients. Further research involving larger groups is essential to determine if our results are consistent across diverse patient populations. Additionally, in terms of neuropsychological examination, we only utilized the MMSE and MoCA scales to assess the presence of cognitive impairment. While these scales provide valuable insights, they may not comprehensively detect all aspects of cognitive profile, particularly among younger patients. What’s more, there was a lack of longitudinal follow-up of functional imaging performance. It is essential to explore alterations in brain microstructure and substance metabolism to understand the pathogenesis of this condition better.

## Conclusion

In conclusion, we summarized the clinical and imaging manifestations of 40 NIID patients. Cognitive impairment was the most prominent clinical feature, while some patients initially presented with episodic symptoms. High signal intensity in the corticomedullary junction area on DWI was the most typical imaging manifestation, and we demonstrated four patterns of its evolution with disease progression. Lesions in the corpus callosum and cerebellum may be early characteristic indicators of NIID. In addition, we observed in detail the changes in brain structure and function caused by the disease using multimodal MRI sequences, which helped to improve the understanding of the imaging characteristics and the pathophysiological substance of the disease.

## Electronic supplementary material

Below is the link to the electronic supplementary material.

Supplementary Material 1

## Data Availability

All relevant data are available from the corresponding authors.

## Abbreviations

NIID: Neuronal intranuclear inclusion disease
DWI: Diffusion-weighted imaging
T1-FLAIR: T1-weighted fluid-attenuated inversion recovery
T2WI: Fast spin‒echo T2-weighted imaging
FLAIR: Fluid-attenuated inversion recovery
DTI: Diffusion tensor imaging
TBSS: Tract-based spatial statistical analysis
MRS: Magnetic resonance spectroscopy
VOI: Volume of interest
LC-Model: Learning-compression model
CSF: Cerebrospinal fluid
PCA: Principal component analysis
MMSE: Mini-mental state examination
MoCA: Montreal cognitive assessment
