# Supplementary material for: Clinical and multimodal imaging features of adult-onset neuronal intranuclear inclusion disease
Source: Neurol Sci. 2024 Jul 18;45(12):5795–805. doi: 10.1007/s10072-024-07699-y (PMC11554744; doi:10.1007/s10072-024-07699-y)
Supplement: Supplementary file 1 — Supplementary Material 1 [file 10072_2024_7699_MOESM1_ESM.docx]

**Methods**

**Imaging protocols**

DTI data underwent a series of preprocessing steps, including format conversion, correction for head motion and eddy currents, and brain extraction[1]. Subsequently, each participant's DTI parameters, including fractional anisotropy (FA) and mean diffusivity (MD) values, were computed using the “dtifit” command within the FSL software (http://www.fmrib.ox.ac.uk/fsl). Tract-based spatial statistics (TBSS) were then applied for statistical analysis and processing. Initially, FA images underwent nonlinear registration to the FMRIB58_FA standard space, from which an average FA image was created. Then, a threshold of 0.2 was applied to generate an average FA skeleton, reducing grey matter influence[2]. FA maps were projected onto this skeleton, producing skeletonized FA maps. White matter fiber skeletons derived from TBSS were utilized as the masking framework for voxel-wise statistical evaluations. Significance was ascertained using a two-tailed P value of less than 0.05, adjusted for family-wise error (FWE) using the threshold-free cluster enhancement (TFCE) approach within the FSL software[3]. The same procedures were applied to MD values, with differences assessed through permutation tests.

**Supplemental Table 1.** Summary of sample sizes and main findings for the examination with NIID patients

| Examination type | Number of  patients | Main findings |
| --- | --- | --- |
| Clinical information | 40 | Main clinical manifestations can be classified as cognitive impairment, movement disorders, limb weakness, paroxysmal symptoms, autonomic dysfunction, and other nonspecific symptoms |
| Neuropsychological assessment |  |  |
| MMSE (score) | 28 | 21 (6-29) ^a^ |
| MoCA (score) | 28 | 14 (4-28) ^a^ |
| NOTCH2NLC GGC repeat size | 24 | 110.5 (91-191) ^a^ |
| Skin biopsy | 19 | Seventeen patients exhibited eosinophilic intranuclear inclusions in the fibroblasts and sweat gland cells |
| Routine MRI |  |  |
| T1WI | 40 | Curvilinear high signal in the corticomedullary junction area on DWI;  Diffuse white matter hyperintensity;  Cerebral atrophy; |
| T2WI | 40 |  |
| T2-FLAIR | 40 |  |
| DWI | 40 |  |
| Follow-up MRI ^b^ | 19 | Four main dynamic patterns of change on DWI |
| Advanced MRI |  |  |
| 3D-T1WI | 21 ^c^ | Significant premature brain aging |
| DTI | 21 ^c^ | Extensive thinning of white matter fibers |
| ^1^H MRS | 5 ^d^ | No significant abnormal metabolism |

^a^ Medians (ranges); ^b^ The patients underwent two or more MRI scans, with follow-up ranging from two to five years; ^c^ Twenty healthy individuals matched for age and sex were collected as the control group; ^d^ Five healthy individuals matched for age and sex were collected as the control group.

**Supplemental Table 2.** Demographic information of patients with NIID and health controls

| Variables | NIID (N = 21) | HC (N = 20) | | P value | |
| --- | --- | --- | --- | --- | --- |
| Gender ratio (male/female) | 6/15 | | 5/15 | | 0.876 |
| Age (years) | 61.0 ± 5.96 | | 64.05 ± 4.01 | | 0.070 |
| MMSE (scores) | 24 (14-29) | | 30 (26-30) | | < 0.001 |
| MoCA (scores) | 16 (9-28) | | 27 (25-29) | | < 0.001 |

Data are presented as mean ± standard or median (range).

HC: Health Controls; MMSE: Mini-Mental State Examination; MoCA: Montreal Cognitive Assessment.


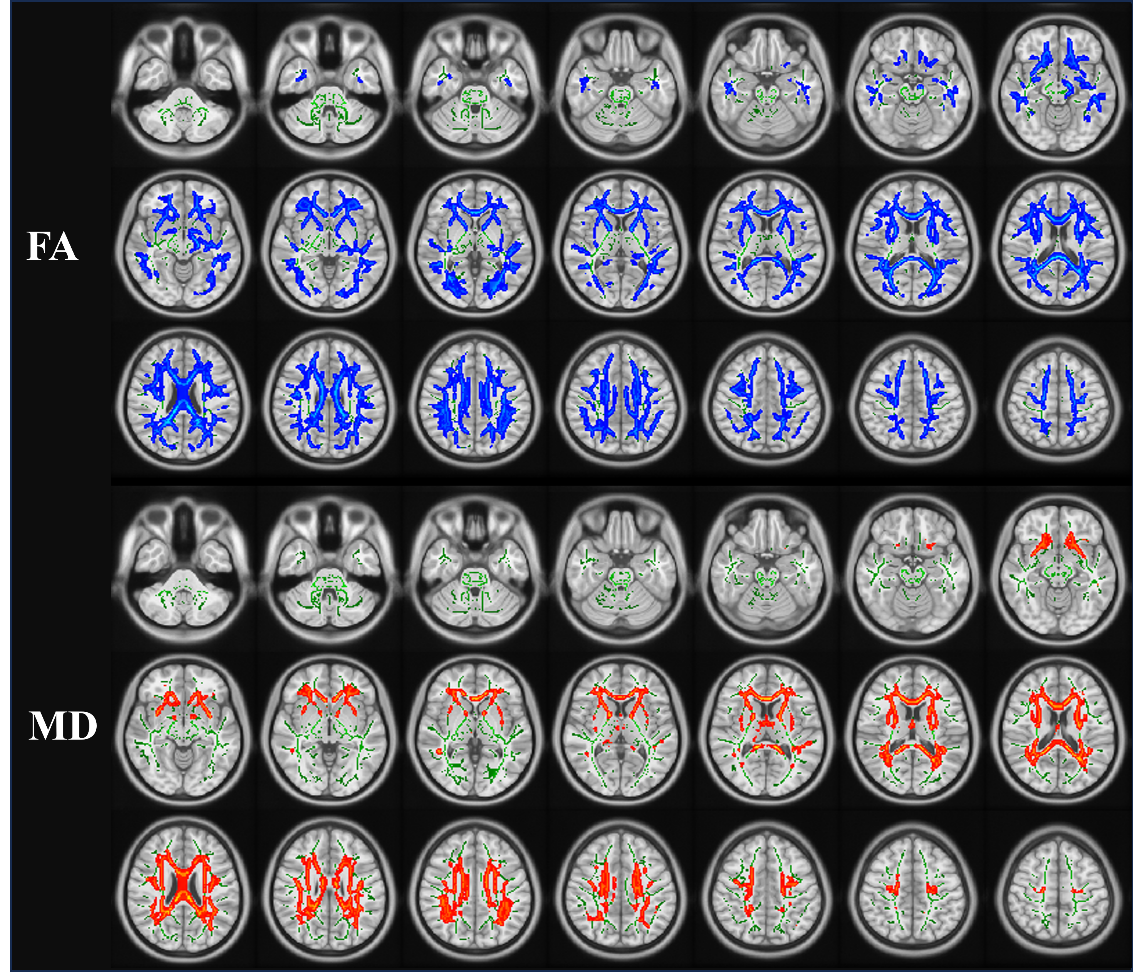


**Supplemental Fig. 1** Tract-based spatial statistics (TBSS) results of DTI metrics between neuronal intranuclear inclusion disease (NIID) patients and healthy controls (HC). Green represents the white matter fiber skeleton structure. Areas highlighted in blue indicate reduced fractional anisotropy (FA) values in the white matter fiber bundles of NIID patients when compared to healthy controls, while regions in red signify increased mean diffusivity (MD) values in NIID patients. All observed differences are statistically significant, with P values less than 0.05 after Family-Wise Error (FWE) correction.


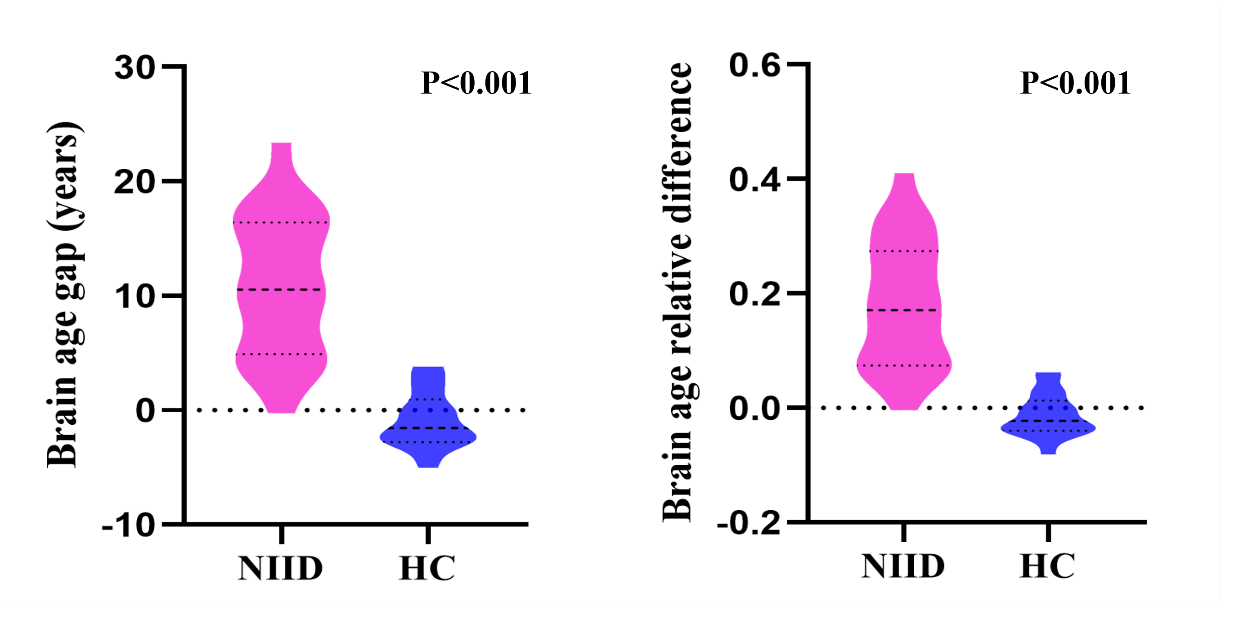


**Supplemental Fig. 2** The difference of brain age between neuronal intranuclear inclusion disease (NIID) patients and healthy controls (HC).

**References:**

1. Jenkinson M, Beckmann C F, Behrens T E et al (2012) FSL. NeuroImage 62:782-790

2. Taylor P A, Alhamud A, van der Kouwe A et al (2016) Assessing the performance of different DTI motion correction strategies in the presence of EPI distortion correction. Hum Brain Mapp 37:4405-4424

3. Winkler A M, Ridgway G R, Webster M A et al (2014) Permutation inference for the general linear model. NeuroImage 92:381-397
